# The neoadjuvant esophageal score: a prognostic tool for predicting survival and postoperative complications in esophageal squamous cell carcinoma

**DOI:** 10.3389/fimmu.2025.1706548

**Published:** 2026-01-02

**Authors:** Hao Chen, Xuan Huang, Cheng Huang, Qichang Xie, Chun Chen, Bin Zheng, Yuxing Lin, Renjie Huang, Li Cheng, Zhang Yang, Chi Xu

**Affiliations:** 1 Department of Thoracic Surgery, Fujian Medical University Union Hospital, Fuzhou, China; 2 Key Laboratory of Cardio-Thoracic Surgery (Fujian Medical University), Fujian Province University, Fuzhou, China; 3 Clinical Research Center for Thoracic Tumors of Fujian Province, Fuzhou, China; 4 National Key Clinical Specialty of Thoracic Surgery, Fuzhou, China; 5 Department of Cardiology, Shengli Clinical Medical College of Fujian Medical University, Fujian Provincial Hospital, Fuzhou University Affiliated Provincial Hospital, Fuzhou, China; 6 Health Management Department, Fuzhou University Affiliated Provincial Hospital, Fuzhou, China; 7 Department of Thoracic Surgery, Fuzhou Pulmonary Hospital of Fujian, Fuzhou, China; 8 Department of Thoracic Surgery, Second Hospital of Zhangzhou, Zhangzhou, China

**Keywords:** esophageal squamous cell carcinoma, neoadjuvant therapy, prognostic score, overall survival, postoperative complications

## Abstract

**Background:**

Esophageal squamous cell carcinoma (ESCC) remains a major global health challenge, and reliable prognostic markers. We developed and validated the Neoadjuvant Esophageal (NAE) score, derived from treatment response, to predict overall survival (OS) and postoperative complications in locally advanced ESCC.

**Methods:**

We retrospectively analyzed 411 patients with ESCC from four hospitals who underwent esophagectomy following neoadjuvant therapy between July 2013 and November 2020. Patients were stratified into low-, intermediate-, and high-score groups. OS was assessed using Kaplan–Meier and Cox regression analyses, and predictive models were constructed with nomograms. Model performance was evaluated using concordance index (C-index), ROC curves, calibration plots, and decision curve analysis.

**Results:**

The NAE score effectively stratified survival outcomes (5-year OS, *p* < 0.001), with higher scores indicating worse prognosis. Adjuvant therapy provided a significant OS benefit only in the high-NAE group (*p* = 0.044), but not in the low- or intermediate-score groups. Multivariable Cox analysis confirmed the NAE score, perineural invasion, lymphovascular invasion, and neutrophil count as independent prognostic factors. A survival nomogram incorporating these variables demonstrated strong discrimination (C-index=0.742) and superior predictive accuracy compared with TNM staging (AUC 0.673–0.835 *vs*. 0.618–0.725). In addition, a complication-prediction nomogram integrating NAE score, surgical approach, and alcohol consumption reliably predicted major postoperative complications (C-index=0.789).

**Conclusion:**

The NAE score is a robust prognostic tool for patients with locally advanced ESCC, capable of predicting survival, guiding adjuvant therapy, and estimating risk of severe postoperative complications. Its integration into clinical practice could refine risk stratification and support personalized treatment strategies, with prospective validation warranted.

## 1 Introduction

Esophageal cancer (EC) is the seventh most common malignancy and the sixth leading cause of cancer-related death worldwide with esophageal squamous cell carcinoma (ESCC) accounts for over 90% of cases (1). Despite advances in multimodal therapy and surgical techniques, long-term survival remains poor, underscoring the need for more reliable prognostic tools (2).

Neoadjuvant systemic therapy has improved outcomes in locally advanced gastrointestinal malignancies, including EC. Several randomized trials have demonstrated that perioperative or neoadjuvant chemotherapy and chemoradiotherapy enhance survival, particularly with regimens incorporating paclitaxel, cisplatin, and fluoropyrimidines (3, 4). Importantly, the degree of pathological response to neoadjuvant therapy has emerged as a powerful prognostic factor across tumor types (5, 6).

In rectal cancer, he Neoadjuvant Rectal (NAR) score was developed to quantify changes in TNM stage before and after neoadjuvant therapy and has been validated as a surrogate endpoint for overall survival (7, 8). This scoring system not only predicts long-term outcomes but also informs decisions regarding adjuvant therapy, potentially sparing patients from unnecessary treatment (9). In ESCC, pathological response is likewise associated with long-term outcomes (10, 11). However, no standardized scoring system exists to quantify treatment response and translate it into prognostic information. Current tools such as tumor regression grade (TRG) and ypN status have limitations, including variability in assessment and inability to guide adjuvant therapy.

Applying a similar principle to ESCC, we sought to develop a novel prognostic index, the Neoadjuvant Esophageal (NAE) score, to quantify treatment response and improve survival prediction. This study aimed to validate the prognostic value of the NAE score in patients with locally advanced ESCC undergoing neoadjuvant therapy and surgery, and to evaluate its utility in guiding adjuvant therapy and predicting postoperative complications.

## 2 Materials and methods

### 2.1 Study design and ethics

This multicenter retrospective study was conducted with approval from the Ethics Committee of Fujian Medical University Union Hospital (as the leading center), with reciprocal approvals from the participating hospitals. Written informed consent was obtained from all participants. The study enrolled adult patients who underwent total esophagectomy at one of the four participating hospitals (Fujian Medical University Union Hospital, Fuzhou University Affiliated Provincial Hospital, Fuzhou Pulmonary Hospital, and second Hospital of Zhangzhou) between July 2013 and November 2020.

### 2.2 Participant selection

Inclusion criteria were: (1) histopathologically confirmed esophageal squamous cell carcinoma (ESCC) post-surgery; (2) receipt of neoadjuvant therapy prior to surgery; (3) presence of a surgically resectable tumor lesion following neoadjuvant therapy; (4) presentation as a single primary tumor lesion. Exclusion criteria were: (1) presence of pulmonary infection within one month preoperatively, severe chronic obstructive pulmonary disease, pulmonary bullae, respiratory or cardiac failure, hyperglycemia, acute or chronic renal insufficiency, significant malnutrition, active inflammation, thrombotic disorders, or hepatic failure; (2) diagnosis of multiple primary malignancies; (3) missing preoperative or postoperative laboratory data or medical records; (4) patients who refused follow-up or were lost to follow-up postoperatively; (5) patients with missing interval data between neoadjuvant therapy and surgery, or an interval exceeding 6 weeks.

During the study period (2013–2020), neoadjuvant immunotherapy was not standard of care for ESCC in China and was not available at our participating centers. All patients received either neoadjuvant chemotherapy (NCT) or chemoradiotherapy (NCRT) based on platinum-fluorouracil regimens; none received immune checkpoint inhibitors or other immunotherapeutic agents.

After applying these selection criteria, we adapted the scoring formula established by George et al. for rectal adenocarcinoma to propose the Neoadjuvant Esophageal (NAE) score. This score is determined by the post-treatment nodal status (ypN) and the pre-treatment (cT) and post-treatment (ypT) T stages. It is calculated as follows:

NAE Score = [(5 × ypN) - 3 × (cT - ypT) + 12]²/9.61

The cohort spans 2013–2020, during which neoadjuvant treatment strategies evolved substantially. Rather than a uniform protocol, patients received heterogeneous regimens including: (1) standard neoadjuvant chemotherapy (NCT) with cisplatin/fluorouracil or paclitaxel/platinum; (2) neoadjuvant chemoradiotherapy (NCRT) with 40–50.4 Gy concurrent with weekly chemotherapy; and (3) modified or institution-specific protocols with variable drug combinations. A meaningful stratified analysis by specific regimen was not feasible due to >15 distinct treatment variations and insufficient sample sizes within individual subgroups (median n=18, range 3–87). This heterogeneity reflects real-world practice patterns but precludes formal sensitivity analysis.

**Figure 1:**
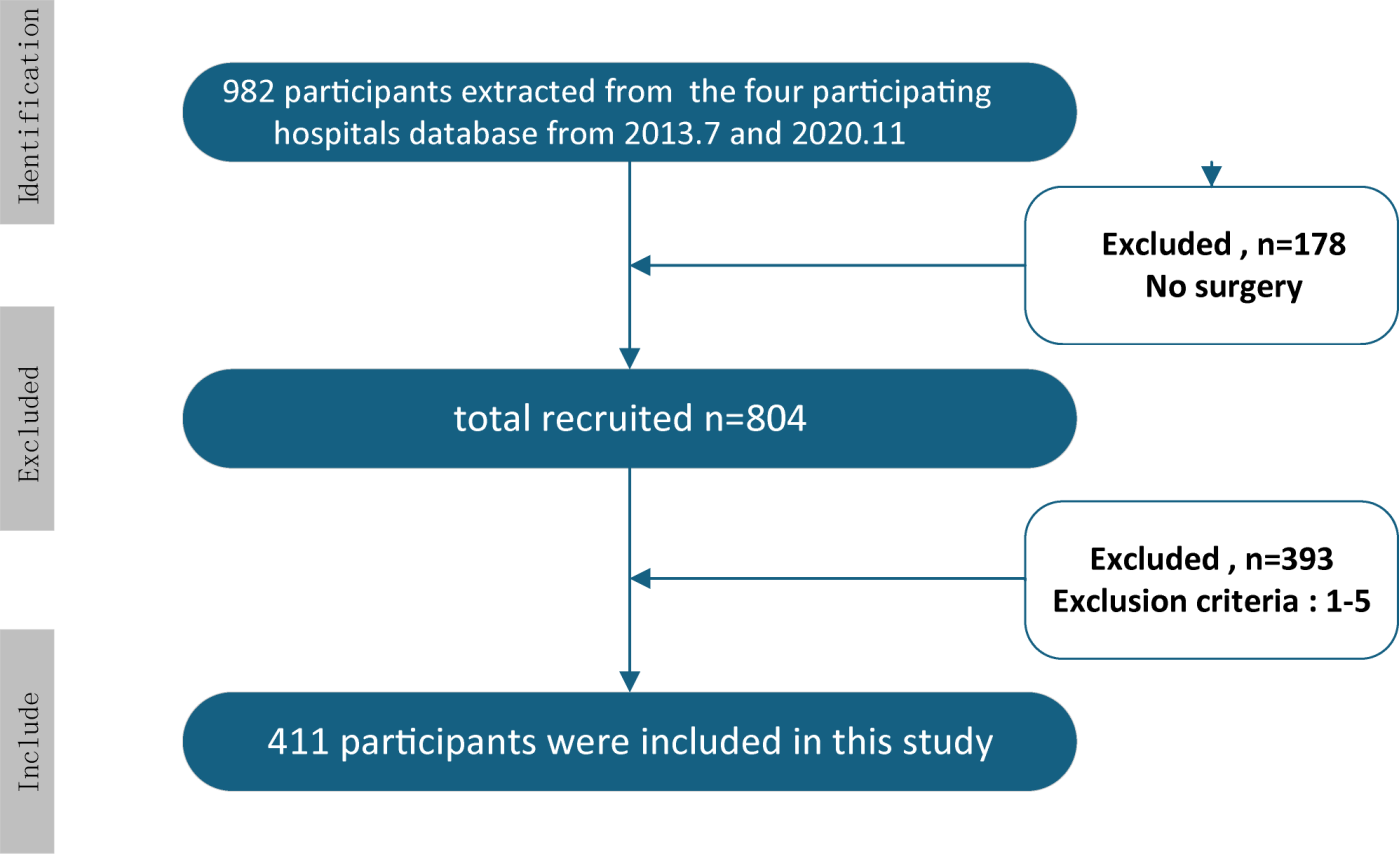
Patient flow diagram showing the screening, exclusion and inclusion process that yielded the final cohort of 411 patients with locally advanced esophageal squamous cell carcinoma who received neoadjuvant therapy followed by radical esophagectomy.

Our study initially identified 982 patients diagnosed with ESCC from the four participating hospitals. During screening, a significant subset of this cohort (n=178) was deemed unsuitable for surgical intervention due to the advanced stage of their ESCC. Following this initial screening, 804 patients remained as potential candidates. To ensure scientific rigor and reliability, we applied the stringent inclusion and exclusion criteria detailed above. After this rigorous refinement process, 411 patients with locally advanced ESCC who underwent surgery following neoadjuvant therapy were ultimately included in the study (**Figure 1**).

### 2.3 Operational definitions

All minimally invasive procedures were performed by senior thoracic surgeons from the participating departments, each with extensive experience in minimally invasive esophagectomy (MIE).

The decision regarding postoperative discharge was based on the assessment of the supervising physician and the fulfillment of the following criteria: (1) optimal overall health, with adaptation to nutritional fluids and restoration of intestinal function; (2) uneventful recovery from surgical stress, with effective pain control; (3) normal body temperature, absence of positive findings on chest and abdominal examination, and laboratory findings within the normal range or close to it; (4) early discharge from bed activities, leading to improved ability to perform daily activities after discharge; (5) no signs of wound infection, and all drains were removed; (6) postoperative follow-up CT indicating good anastomotic healing. The primary outcome measure was LOS, calculated from the day of surgery to discharge.

### 2.4 Measured variables

Preoperative demographic and clinical data were collected, including blood samples, age, sex, body mass index (BMI), overall survival (OS), length of hospital stay (LOS), smoking history (cumulative lifetime smoking ≥100 cigarettes), alcohol consumption history (consumed alcohol at least once in the past 12 months), preoperative tumor imaging, tumor location, postoperative TNM staging, presence or absence of perineural and lymphovascular invasion, and postoperative complications. Fasting peripheral blood samples were collected 1 to 7 days prior to surgical intervention. Normal BMI was defined as 18.5–23.9 kg/m². Established reference intervals for blood biomarkers included: neutrophil count (1.5–7.0 × 10^9^/L), lymphocyte count (0.8–4.0 × 10^9^/L), monocyte count (0.12–0.8 × 10^9^/L), platelet count (100–300 × 10^9^/L), carcinoembryonic antigen (CEA; 0–5 ng/mL), and carbohydrate antigen 19-9 (CA19-9; 0–37 U/mL).

### 2.5 Statistical analysis

Based on the NAE score, patients were stratified into low (<8 points), intermediate (8–16 points), and high (>16 points) groups, with lower scores indicating a more favorable response to neoadjuvant therapy. These cut-off values were directly adopted from the original Neoadjuvant Rectal (NAR) score, as the NAE score formula was adapted from this validated system. The NAR score cut-offs were established based on extensive validation in rectal cancer cohorts and have been widely accepted in the literature. Given the analogous mathematical structure and clinical application of our NAE score (i.e., quantifying treatment response based on T/N downstaging), we maintained these identical thresholds to ensure consistency with the established literature and facilitate comparability across studies. In our cohort, these cut-offs effectively discriminated survival outcomes (log-rank p<0.001), supporting their applicability in esophageal squamous cell carcinoma. Differences in categorical variables between groups were assessed using conditional logistic regression, while continuous variables were analyzed using linear mixed-effects models. To adjust for confounders, propensity scores were calculated using age, sex, BMI, Charlson Comorbidity Index, clinical T stage (cT), clinical N stage (cN), surgical approach, number of lymph nodes dissected, and adjuvant therapy as covariates. Patients were matched using 1:1:1 nearest-neighbor matching with a caliper width of 0.05 standard deviations (SD). Following matching, survival analysis was performed using the Kaplan-Meier method. Differences in OS between patients who received adjuvant therapy and those who did not were compared within each NAE subgroup.

The secondary objective was to construct predictive models for OS and postoperative complications: a Cox proportional hazards regression model was developed for OS prediction, and a logistic regression model was developed for complication prediction. Model performance was evaluated using decision curve analysis (DCA), time-dependent area under the curve (time-AUC), calibration curves, and receiver operating characteristic (ROC) curves. All analyses were performed using IBM SPSS Statistics version 27.0 (IBM Corp., Armonk, NY, USA) and R version 4.5.0 (R Foundation for Statistical Computing, Vienna, Austria). Specific R packages used for key analyses included: MatchIt (version 4.5.0) for propensity score matching, rms (version 6.7-0) for nomogram construction and calibration, survival (version 3.5-5) for Cox regression analyses, and pROC (version 1.18.4) for ROC curve analysis. The significance level was set at p < 0.05.

A complete case analysis was performed in this study. As specified in the exclusion criteria (Section 2.2), patients with missing preoperative or postoperative laboratory data, medical records, or key pathological variables (cT, ypT, ypN) were excluded from the analysis. No imputation methods were employed. Among the 411 included patients, all had complete data for the variables used in the final multivariable models (NAE score, perineural invasion, lymphovascular invasion, neutrophil count, surgical approach, and alcohol consumption). The propensity score matching also utilized only complete cases, ensuring that the matched cohorts had no missing covariate information.

## 3 Results

### 3.1 Baseline characteristics and treatment analysis of ESCC

A total of 411 patients with locally advanced ESCC were included in the final analysis. The median age was 60 years (range 42–77), and the majority were male (79.1%). Most patients had a Charlson Comorbidity Index (CCI) score of 0 (62.5%) and tumors located in the mid-esophagus (59.9%).Clinically, cT3 (41.1%) and cN2 (38.4%) were the most frequent stages at diagnosis. Postoperatively, the most common pathological stages were ypT3 (49.6%) and ypN0 (49.1%). Pathological complete response (pCR; ypT0N0) was achieved in 43 patients (10.5%).The McKeown procedure was the predominant surgical approach (58.2%), with a median of 33 lymph nodes retrieved per patient. Regarding adjuvant therapy, 79.8% of patients received postoperative systemic treatment: radiation alone (29.0%), chemotherapy alone (20.7%), or combined chemoradiotherapy (30.2%).When stratified by NAE score, 71 patients (17.3%) were classified as low, 131 (31.9%) as intermediate, and 209 (50.8%) as high score. Detailed clinicopathological and treatment characteristics are summarized in **Table 1**.

**Table 1:** Description of clinical and demographic characteristics of the selected patients.

N	Subgroup/Statistics	411
Age	Mean ± SD, median	59.7 ± 7.85
Sex	Male	325(79.1%)
	Female	86(20.9%)
Charlson score	0	257(62.5%)
	1	106(25.8%)
	2	32(7.8%)
	3+	16(3.9%)
Location	up	43(10.6%)
	mid	246(59.9%)
	low	122(29.7%)
Clinical T stage	cT1	35(8.5%)
	cT2	101(24.6%)
	cT3	169(41.1%)
	cT4	106(25.8%)
Clinical N stage	cN0	83(20.2%)
	cN1	102(24.8%)
	cN2	158(38.4%)
	cN3	68(16.5%)
Surgery	Ivor-Lewis	172(41.8%)
	McKeown	239(58.2%)
Pathologic T stage	ypT0	60(14.6%)
	ypT1	58(14.1%)
	ypT2	74(18.0%)
	ypT3	204(49.6%)
	ypT4	15(3.6%)
Pathologic N stage	ypN0	202(49.1%)
	ypN1	123(29.9%)
	ypN2	64(15.6%)
	ypN3	22(5.4%)
Number of retrieved nodes	Mean ± SD, median	33.4 ± 8.8
Pathologic complete response	ypT0 ypN0	43(10.5%)
Neoadjuvant esophageal score	Low	71(17.3%)
	Intermediate	131(31.9%)
	High	209(50.9%)
Adjuvant therapies	No	83(20.2%)
	Adjuvant radiation	119(29.0%)
	Adjuvant chemotherapy	85(20.7%)
	Adjuvant chemotherapy+radiotherapy	124(30.2%)

### 3.2 Survival differences by NAE score stratification and impact of adjuvant therapy

**Figure 2:**
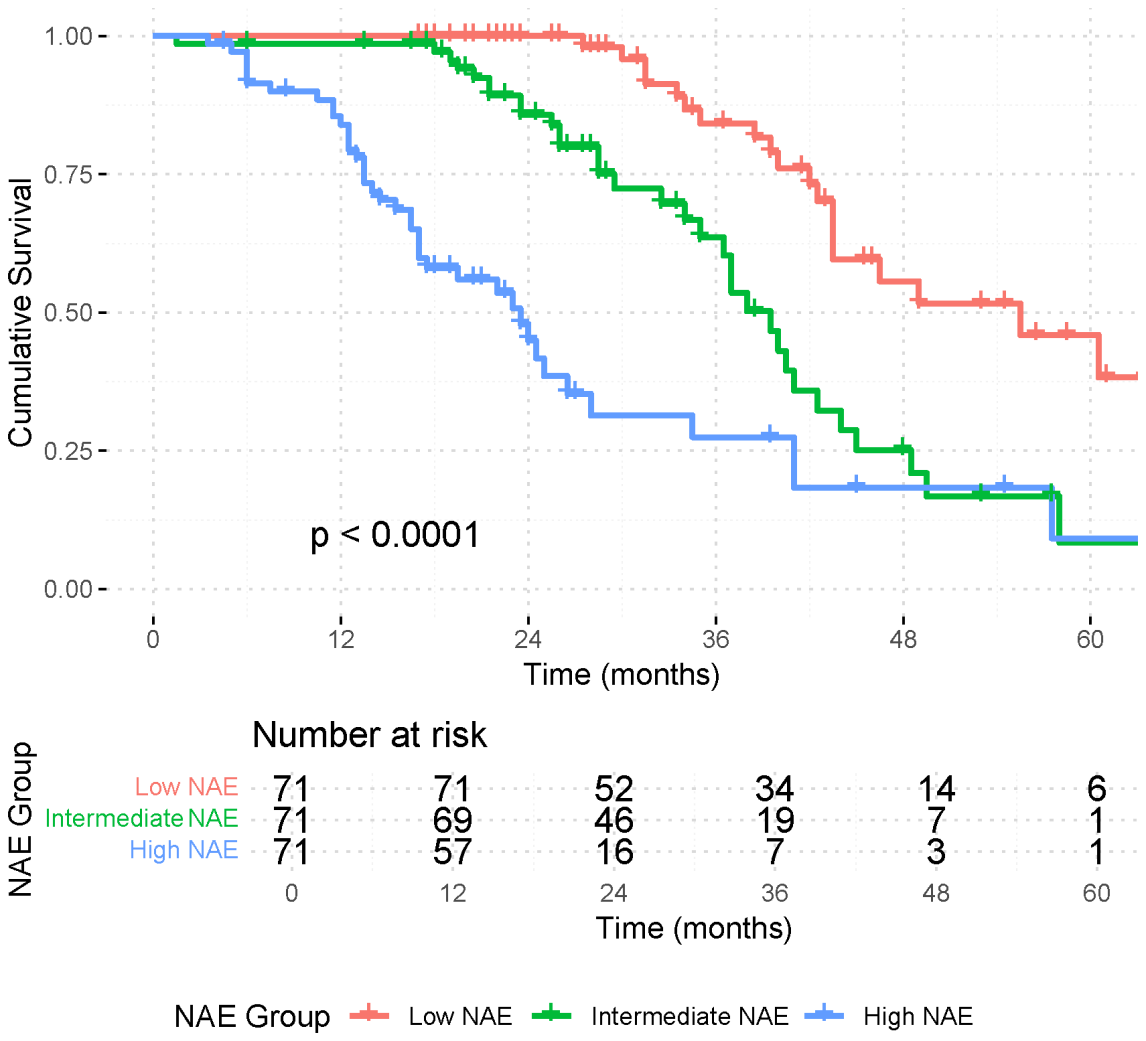
Kaplan–Meier graph for overall survival in the matched patients by NAE score. Number at risk is shown below the plot.

Following stratification by NAE score, patients were classified into low (n=71, 17.3%), intermediate (n=131, 31.9%), and high (n=209, 50.8%) groups. Before matching, baseline differences were observed across Charlson Comorbidity Index, clinical stage, surgical approach, lymph node yield, and distribution of adjuvant therapy (**Table 2**). After 1:1:1 propensity score matching, 71 patients remained in each group, achieving well-balanced characteristics (**Table 3**). Kaplan–Meier analysis demonstrated significantly different OS among the three groups after matching (*p* < 0.001; **Figure 2**). Patients with higher NAE scores consistently had shorter survival, confirming the score’s prognostic value. In contrast, length of hospital stay (LOS) did not significantly differ among the groups (**Supplementary Figure S1**).

**Table 2:** Baseline characteristics before and after propensity score matching.

Characteristics	Subgroup/Statistics	Low NAE (n=71)	Intermediate NAE (n=131)	High NAE (n=209)	P	Low NAE (n=71)	Intermediate NAE (n=71)	High NAE (n=79)	P
		Before matching				After matching			
Age	Mean ± SD	60.6 ± 8.2	59.7 ± 7.5	59.5 ± 8.0	0.599	60.6 ± 8.2	60.8 ± 7.6	60.3 ± 7.7	0.932
BMI	Mean ± SD	21.5 ± 2.8	22.2 ± 2.9	21.8 ± 3.0	0.261	21.5 ± 2.8	21.9 ± 3.1	21.7 ± 3.0	0.701
Sex	Male	56(78.9%)	104(79.4%)	165(78.9%)	0.994	56(78.9%)	54(76.1%)	62(87.3%)	0.208
	Female	15(21.1%)	27(20.6%)	44(21.1%)		15(21.1%)	17(23.9%)	9(12.7%)	
Charlson score	0	52(73.2%)	92(70.2%)	113(54.1%)	0.031	52(73.2%)	56(78.9%)	48(67.6%)	0.565
	1	14(19.7%)	28(21.4%)	64(30.6%)		14(19.7%)	10(14.1%)	17(23.9%)	
	2	3(4.2%)	7(5.3%)	22(10.5%)		3(4.2%)	3(4.2%)	4(5.6%)	
	3+	2(2.8%)	4(3.1%)	10(4.8%)		2(2.8%)	2(2.8%)	2(2.8%)	
Clinical T stage	1	0(0.0%)	12(9.2%)	23(11.0%)	< 0.001	0(0.0%)	0(0.0%)	0(0.0%)	0.402
	2	9(12.7%)	25(19.1%)	67(32.1%)		9(12.7%)	4(5.6%)	2(2.8%)	
	3	29(40.8%)	61(46.6%)	79(37.8%)		29(40.8%)	34(47.9%)	33(46.5%)	
	4	33(46.5%)	33(25.2%)	40(19.1%)		33(46.5%)	33(46.5%)	36(50.7%)	
Clinical N stage	0	25(35.2%)	28(21.4%)	30(14.4%)	< 0.001	25(35.2%)	17(23.9%)	7(9.9%)	0.318
	1	8(11.3%)	36(27.5%)	58(27.8%)		8(11.3%)	25(35.2%)	26(36.6%)	
	2	23(32.4%)	53(40.5%)	82(39.2%)		23(32.4%)	19(26.8%)	29(40.8%)	
	3	15(21.1%)	14(10.7%)	39(18.7%)		15(21.1%)	10(14.1%)	9(12.7%)	
Surgery	Ivor-Lewis	38(53.5%)	59(45.0%)	75(35.9%)	0.023	38(53.5%)	41(57.7%)	33(46.5%)	0.397
	McKeown	33(46.5%)	72(55.0%)	134(64.1%)		33(46.5%)	30(42.3%)	38 (53.5%)	
Retrieved nodes	Mean ± SD	31.9 ± 9.0	32.5 ± 8.7	34.5 ± 8.7	0.044	31.9 ± 9.0	30.6 ± 8.3	32.6 ± 8.6	0.368
Adjuvant therapies	No	16(22.5%)	45(34.4%)	22(10.5%)	< 0.001	16(22.5%)	30(42.3%)	14(19.7%)	0.452
	Adjuvant radiation	15(21.1%)	35(26.7%)	69(33.0%)		15(21.1%)	12(16.9%)	18(25.4%)	
	Adjuvant chemotherapy	23(32.4%)	20(15.3%)	42(20.1%)		23(32.4%)	17(23.9%)	20(28.2%)	
	Adjuvant chemotherapy+radiotherapy	17(23.9%)	31(23.7%)	76(36.4%)		17(23.9%)	12(16.9%)	19(26.8%)	

**Table 3:** Correlations between OS and NAE and other clinicopathological factors.

Characteristics	Univariate analysis		Multivariate analysis	
	Hazard ratio(95%CI)	P-value	Hazard ratio(95%CI)	P-value
Age				
<60	reference			
≥60	1.019(0.768-1.353)	0.894		
Sex				
Female	reference		reference	
male	1.231(0.870-1.742)	0.240	1.182(0.761-1.836)	0.456
BMI				
≤23.9	reference		reference	
>23.9	0.910(0.642-1.290)	0.596	0.660(0.457-0.951)	0.026
Smoke				
No	reference		reference	
Yes	1.255(0.945-1.667)	0.117	1.100(0.737-1.640)	0.641
Drink				
No	reference		reference	
Yes	1.255(0.948-1.662)	0.112	1.293(0.892-1.874	0.174
Adjuvant therapies				
No	reference		reference	
Adjuvant radiation	0.838(0.541-1.298)	0.428	0.501(0.294-0.853)	0.011
Adjuvant chemotherapy	1.245(0.803-1.931)	0.327	1.292(0.775-2.154)	0.326
Adjuvant chemotherapy+radiotherapy	1.240(0.821-1.874)	0.306	0.790(0.481-1.299)	0.353
ypT				
0	reference		reference	
1	1.675(0.856-3.276)	0.132	0.849(0.393-1.833)	0.677
2	2.755(1.516-5.006)	<0.001	0.898(0.431-1.873)	0.774
3	4.521(2.632-7.767)	<0.001	0.956(0.471-1.941)	0.902
4	3.743(1.634-8.572)	0.002	1.124(0.756-1.548)	0.298
ypN				
0	reference		reference	
1	1.909(1.367-2.665)	<0.001	0.665(0.362-1.223)	0.19
2	2.606(1.784-3.806)	<0.001	0.768(0.392-1.505)	0.442
3	5.191(2.954-9.122)	<0.001	1.515(0.653-3.512)	0.333
Perineural invasion				
0	reference		reference	
1	2.083(1.516-2.860)	<0.001	1.574(1.100-2.252)	0.013
Lymphovascular invasion				
0	reference		reference	
1	1.802(1.344-2.416)	<0.001	1.471(1.135-1.867)	0.040
Clavien-Dindo				
I-II	reference		reference	
III-V	1.166(0.823-1.651)	0.388	0.907(0.630-1.305)	0.599
Neoadjuvant esophageal score				
Low	reference		reference	
Intermediate	4.714(2.409-9.224)	<0.001	6.478(2.835-14.805)	<0.001
High	10.444(5.471-19.936)	<0.001	20.552(7.623-55.406)	<0.001
Neutrophil count	0.879(0.790-0.978)	0.018	0.840(0.747-0.944)	0.003
Lymphocyte count	0.885(0.707-1.108)	0.288		
Monocyte count	0.422(0.198-0.898)	0.025	0.716(0.319-1.607)	0.418
Platelet count	1.000(0.998-1.002)	0.955		
CEA	0.975(0.919-1.034)	0.393		
CA199	1.000(0.996-1.004)	0.990		
NAE × Age interaction	NA	NA	0.780(0.491-1.253)	0.306*
NAE × Sex interaction	NA	NA	1.361(0.782-2.363)	0.275*

*The interaction between NAE score and age (or sex) was assessed using a Wald z-test (df = 1).

The survival benefit of adjuvant therapy was dependent on NAE score. In the low-NAE group, no OS benefit was observed (*p* = 0.22), though survival curves began to diverge after ~85 months. Similarly, the intermediate-NAE group showed no significant survival difference with adjuvant therapy (*p* = 0.93). In contrast, the high-NAE group experienced a significant OS advantage when adjuvant therapy was administered (*p* = 0.044; **Supplementary Figures S2**-**4**).

These findings indicate that the NAE score not only stratifies long-term survival but also identifies the subgroup of high-risk patients who derive meaningful benefit from adjuvant treatment.

### 3.3 Prognostic factors for patient overall survival

Univariate analysis demonstrated significant associations between overall survival (OS) and ypT stage, ypN stage, perineural invasion, lymphovascular invasion, NAE score, neutrophil count, and monocyte count (all *p* < 0.05; **Table 4**). In the multivariable Cox model, four factors retained independent prognostic value. A higher NAE score was strongly correlated with increased mortality risk (Intermediate *vs*. Low: HR 6.48, 95% CI 2.84–14.81; High *vs*. Low: HR 20.55, 95% CI 7.62–55.41; *p* < 0.001). The presence of perineural invasion (HR 1.57, 95% CI 1.10–2.25; *p* = 0.013) and lymphovascular invasion (HR 1.47, 95% CI 1.14–1.87; *p* = 0.040) were also adverse predictors, whereas a higher neutrophil count was unexpectedly associated with better OS (HR 0.84, 95% CI 0.75–0.94; *p* = 0.003).

**Table 4:** Univariate and multivariate logistic regression analyses assessing the association between clinical factors and postoperative Clavien-Dindo grade ≥III complications, presented as OR (95% CI) and p-value.

Characteristics	Univariate analysis		Multivariate analysis	
	OR(95%CI)	P	OR(95%CI)	P
Age(years)	0.998(0.963-1.035)	0.920	0.991(0.952-1.031)	0.651
BMI(kg/m²)	0.984(0.892-1.085)	0.747	0.969(0.872-1.076)	0.555
Sex				
Female	1.00(Reference)		1.00(Reference)	
Male	1.489(0.776-2.855)	0.231	1.263(0.529-3.011)	0.599
Smoke				
No	1.00(Reference)		1.00(Reference)	
Yes	1.348(0.760-2.391)	0.307	1.119(0.478-2.619)	0.795
Drink				
No	1.00(Reference)		1.00(Reference)	
Yes	1.951(1.081-3.519)	0.026	2.024(0.947-4.324)	0.069
Surgery				
Ivor-Lewis	1.00(Reference)		1.00(Reference)	
McKeown	3.279(1.636-6.570)	<0.001	4.166(1.870-9.278)	<0.001
Number of retrieved nodes (n)	1.008(0.976-1.041	0.637	0.968(0.931-1.007)	0.110
Platelet counts (×10^9^/L)	1.000(0.996-1.005)	0.924	1.000(0.996-1.005)	0.899
Neutrophil counts (×10^9^/L)	1.007(0.884-1.202)	0.937	0.920(0.745-1.136)	0.440
Lymphocyte count (×10^9^/L)	1.117(0.710-1.757)	0.633	1.058(0.642-1.743)	0.826
Monocyte count (×10^9^/L)	1.528(0.437-5.341)	0.507	3.483(0.826-14.689)	0.089
Neoadjuvant esophageal score				
Low	1.00(Reference)		1.00(Reference)	
Intermediate	2.023(0.638-6.412)	0.231	1.873(0.574-6.118)	0.299
High	3.620(1.239-10.573)	0.019	3.275(1.083-9.904)	0.036
NAE × Surgery interaction	NA	NA	NA	0.299*

* means Global likelihood-ratio test for the interaction block (df = 2).

**Figure 3:**
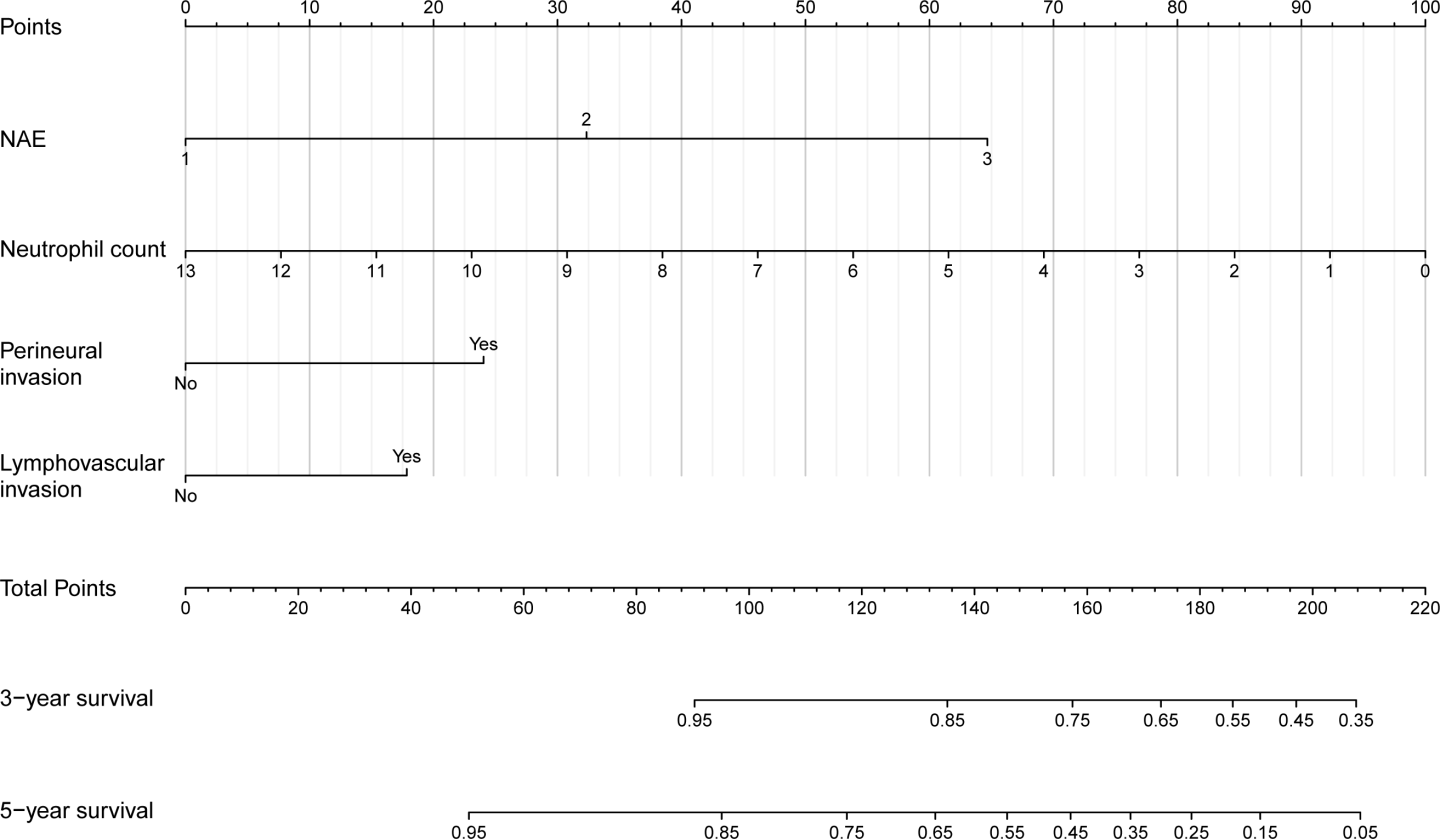
Nomogram to predict OS in patients with locally advanced ESCC undergoing neoadjuvant therapy.

**Figure 4:**
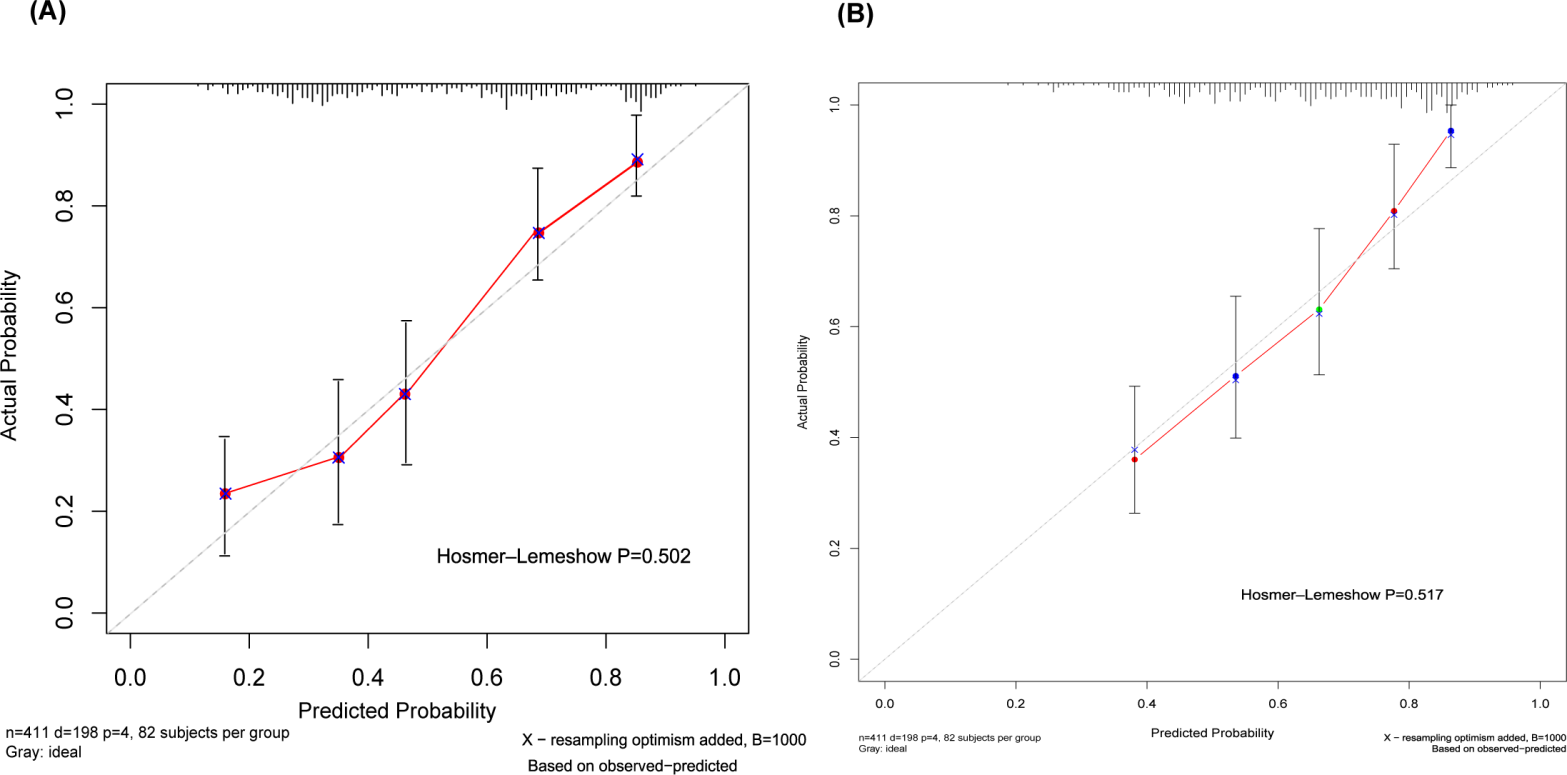
Calibration curves for 3-year OS **(A)** and 5-year OS **(B)** prediction based on nomogram.

Based on these findings, we constructed a prognostic nomogram incorporating the NAE score, perineural invasion, lymphovascular invasion, and neutrophil count (**Figure 3**). The model demonstrated robust discrimination (C-index=0.742, 95% CI 0.694–0.790) and good calibration, with close agreement between predicted and observed 3- and 5-year OS (**Figures 4A, B**). Weighted points were assigned to each variable, and total scores were translated into survival probabilities; notably, patients with scores <120 points had >85% probability of 3-year survival, while those with scores <80 points achieved >85% probability of 5-year survival.

**Figure 5:**
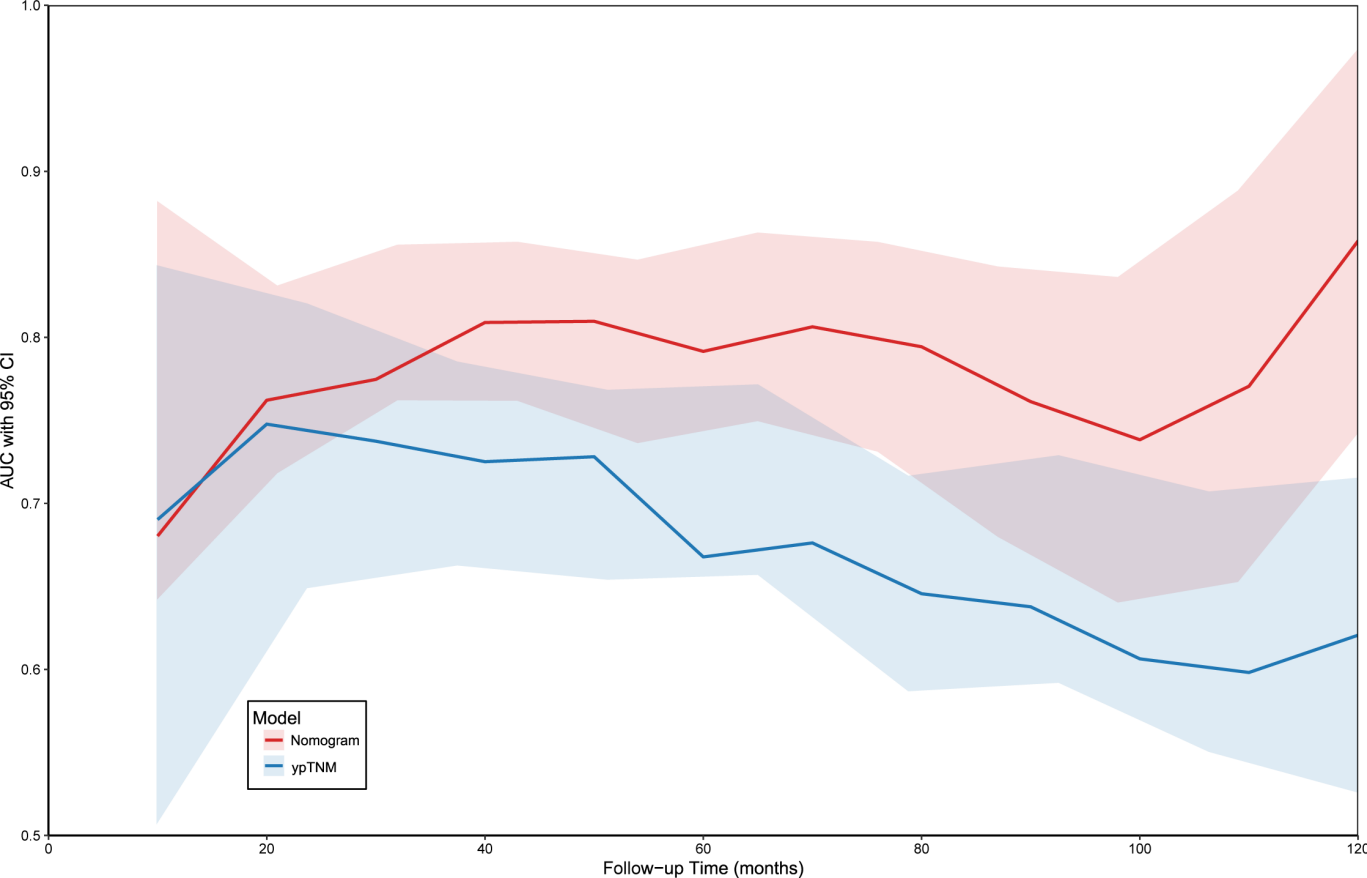
Compare the ability to predict OS with the pathological TNM stage by t-AUC.

Time-dependent ROC analysis confirmed that the nomogram outperformed pathological TNM staging, with an AUC range of 0.673–0.835 compared with 0.618–0.725 for TNM alone (**Figure 5**). Collectively, these results demonstrate that for patients with locally advanced ESCC after neoadjuvant therapy, the nomogram provides significantly superior prognostic accuracy compared with conventional TNM staging.

### 3.4 Predictive factors for postoperative complications

**Figure 6:**
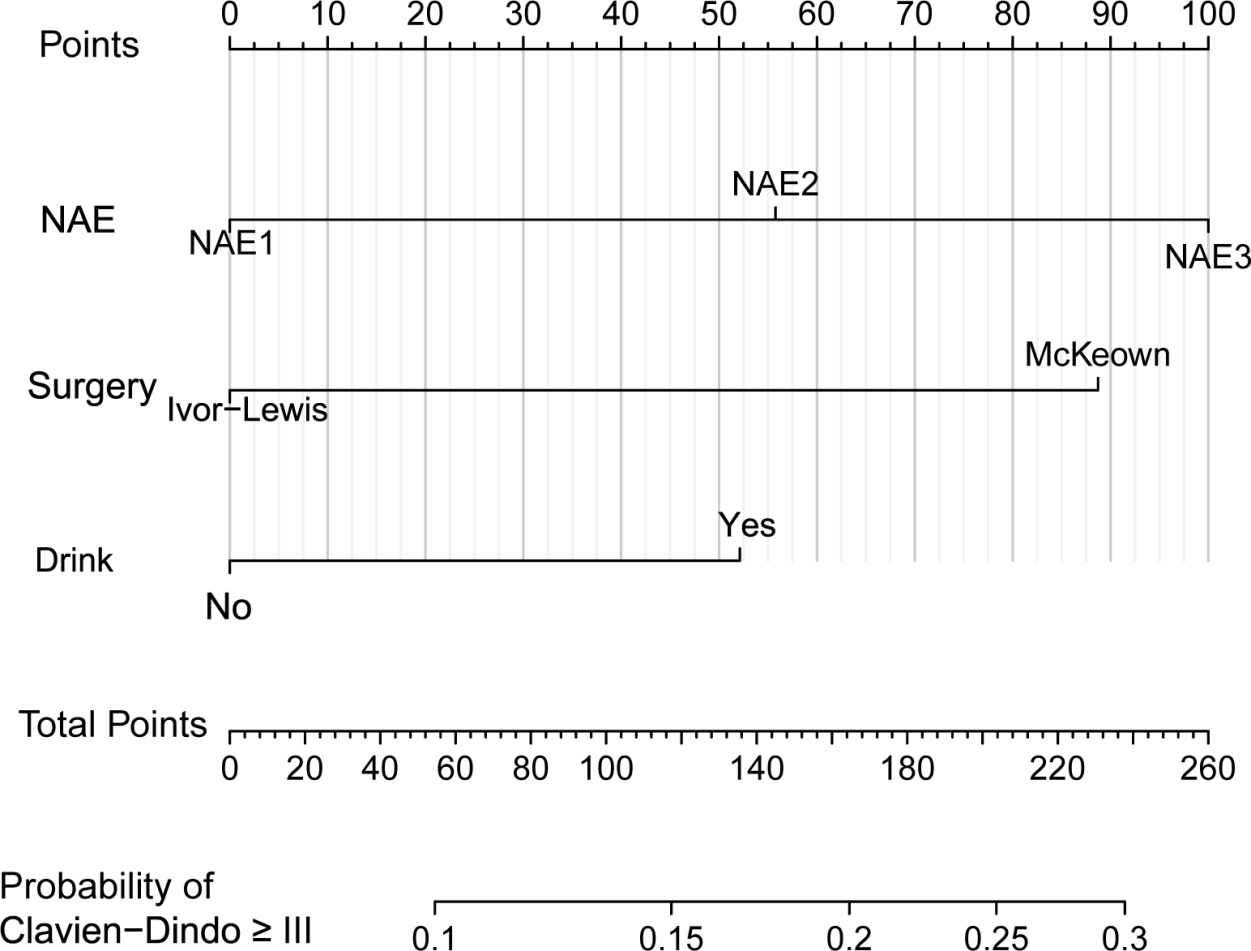
Nomogram to predict Clavien–Dindo grade ≥III complications after surgery.

**Figure 7:**
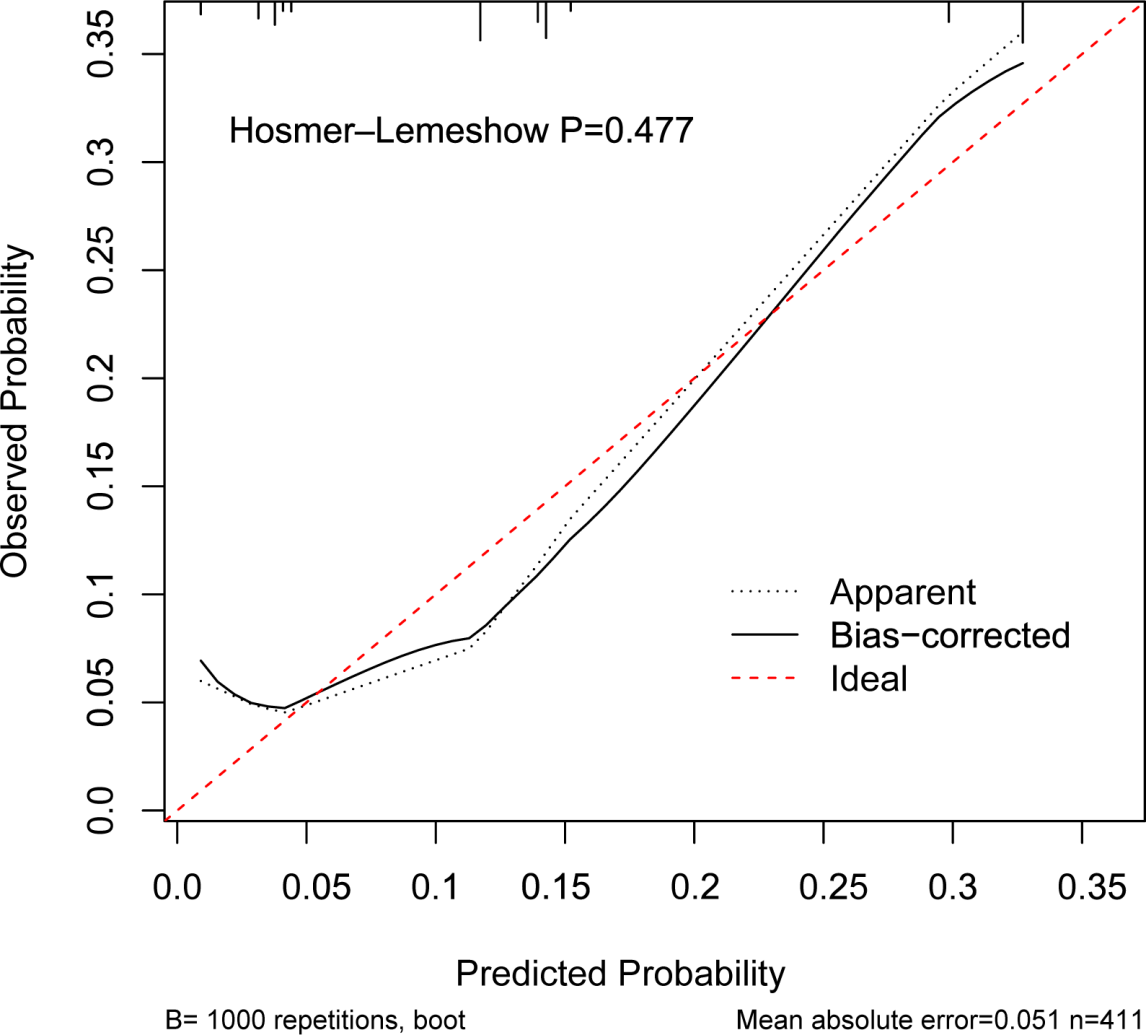
Calibration curves for Clavien–Dindo grade ≥III complications based on nomogram.

Logistic regression analyses identified the NAE score and surgical approach as independent predictors of major postoperative complications (Clavien–Dindo grade ≥III), while alcohol consumption showed a borderline association with increased risk (OR = 2.02, 95% CI 0.95–4.32; *p* = 0.07). A nomogram incorporating these three variables demonstrated strong predictive performance, with a C-index of 0.789 (95% CI 0.738–0.840; **Figure 6**). Calibration analysis (**Figure 7**) confirmed close agreement between predicted probabilities and observed outcomes, and ROC analysis yielded an AUC of 0.789 (95% CI 0.738–0.840; **Supplementary Figures S5**). Decision curve analysis further showed meaningful clinical net benefit within a threshold probability range of 0.2–0.6 (**Supplementary Figures S6**). Collectively, these results support the substantial clinical utility of this model in predicting severe postoperative complications after esophagectomy in patients with ESCC.

## 4 Discussion

In this study, we established and validated the Neoadjuvant Esophageal (NAE) score as a novel prognostic tool for patients with locally advanced ESCC undergoing neoadjuvant therapy followed by esophagectomy. The NAE score effectively stratified survival outcomes, demonstrated superior prognostic accuracy compared with the TNM staging system, and identified patients most likely to benefit from adjuvant therapy. Furthermore, nomograms incorporating the NAE score with additional clinicopathological factors provided reliable predictions for both long-term survival and postoperative complications (12).

Current risk assessment tools in esophageal cancer remain limited (13). The traditional TNM staging system offers only a static snapshot at diagnosis, failing to capture dynamic changes during treatment (14). Although previous studies, such as that by Shen Q et al. (2022), have developed clinical prediction models using non-TNM factors, and others have refined prognosis based on TNM staging, significant gaps persist (15, 16). Tumor regression grade (TRG), widely used to assess neoadjuvant response, suffers from variability due to sampling quality and inter-observer differences, poor reproducibility, and inability to predict benefit from adjuvant therapy (17–19). Postoperative nodal status (ypN), while prognostic, reflects only post-treatment local disease burden without incorporating pretreatment tumor extent (cT). Consequently, existing tools lack dynamic quantification across the pre- to post-treatment continuum and fail to guide adjuvant therapy decisions.

In contrast, the NAE score represents a significant advancement by dynamically integrating pretreatment (cT) and post-treatment (ypT, ypN) pathological data, providing a continuous measure of therapeutic response. Adapted from the concept of the Neoadjuvant Rectal (NAR) score (7), which has been validated as a surrogate endpoint in rectal cancer, the NAE score extends this principle to ESCC. Unlike TRG, which is prone to subjectivity and sampling bias, the NAE score relies on standardized, routinely reported pathological parameters, enhancing its reproducibility and clinical feasibility. Moreover, whereas ypN stage alone offers a static postoperative assessment, the NAE score captures the magnitude of downstaging, offering a more nuanced reflection of tumor biology and treatment efficacy.

A key advantage of the NAE score lies in its ability to inform adjuvant therapy decisions—a capability notably absent in existing systems. Regarding the evolution of chemotherapy regimens, while shifting from CF to DCF has significantly increased pathologic complete response rates, it has also escalated hematologic toxicity (20–22). Although NCCN guidelines recommend postoperative adjuvant therapy (23), effective patient selection tools are lacking. We found that patients with high NAE scores (>16) gained a substantial (>15%) improvement in 5-year overall survival with adjuvant therapy, whereas those with low-intermediate NAE scores showed no significant benefit. This supports the use of the NAE score to inform “step-up/step-down” adjuvant therapy strategies: considering treatment reduction or omission for low-intermediate risk patients to reduce toxicity, while actively recommending it for high-risk patients to improve survival.

Beyond prognosis, we constructed an overall survival-predicting nomogram incorporating the NAE score, perineural invasion, lymphovascular invasion, and neutrophil count—all identified as independent prognostic factors in our Cox analysis. This model demonstrated good predictive accuracy (C-index=0.742) and significantly outperformed traditional pathologic TNM staging in time-dependent AUC analysis. Similarly, recognizing the impact of surgical approach and alcohol consumption on complications, we developed a complication-predicting nomogram (C-index=0.789) that also exhibited strong discrimination and calibration. Interaction analysis confirmed that the NAE score’s performance was consistent across surgical approaches and unaffected by age or sex, supporting its generalizability. This stability contrasts with some existing biomarkers that may exhibit population-specific variability.

Unexpectedly, we also observed that higher peripheral neutrophil counts were independently associated with improved survival—a finding that contrasts with the more commonly reported adverse prognostic role of neutrophil-to-lymphocyte ratios (24) in cancer. This warrants further mechanistic investigation.

This study has several limitations. Our cohort predates the widespread adoption of neoadjuvant immunotherapy for ESCC. Since 2020, immune checkpoint inhibitors (ICIs) combined with chemotherapy or chemoradiotherapy have demonstrated promising efficacy and are increasingly used in clinical practice and trials. The NAE score’s performance in patients receiving immunotherapy remains to be determined. However, given that the score quantifies pathological response (ypT/ypN) irrespective of the neoadjuvant regimen, we hypothesize it may remain valid. The magnitude of downstaging achieved with immunotherapy-based regimens may differ, potentially shifting the score distribution, but the biological principle linking treatment response to prognosis should persist. Prospective validation in immunotherapy-treated cohorts is an important next step to confirm generalizability. This is particularly critical for Frontiers in Immunology readers, as integrating the NAE score into immunotherapy trials could provide a standardized surrogate endpoint for response assessment. First, and most critically, this study lacks an independent external validation cohort, which represents the primary limitation of our work. While our multicenter design (four hospitals) provides internal validation, all centers are within Fujian Province with similar treatment practices. The NAE score requires validation in completely independent cohorts from different geographic regions, ethnic populations, and healthcare systems to establish it as a robust, generalizable biomarker. We are actively collaborating with centers in other provinces for external validation, but these data were not mature at submission. Clinicians should consider the NAE score internally validated but not externally validated at this stage. Second, the study spans 2013-2020, a period of substantial evolution in neoadjuvant regimens and surgical techniques. Our cohort encompasses >15 distinct treatment protocols, including institutional modifications and clinician-individualized therapy. This complexity precluded meaningful stratified analysis due to insufficient power in individual regimen subgroups. While this heterogeneity limits our ability to assess regimen-specific performance, the NAE score’s robust prognostic value across these diverse settings may demonstrate its generalizability. However, we cannot definitively exclude confounding by treatment era. Prospective validation within homogeneous, protocol-defined cohorts is essential, particularly as standardized immunotherapy-based regimens emerge. Finally, although the NAE score was modeled after the NAR score, prospective studies are required to confirm its utility as a surrogate endpoint in clinical trials.

In conclusion, the NAE score is a simple yet powerful prognostic tool that dynamically assesses treatment response, stratifies survival, predicts postoperative complications, and informs adjuvant therapy decisions. Its integration into clinical practice could enhance individualized management and optimize therapeutic strategies. Future multicenter prospective studies are warranted to validate its applicability across diverse populations and treatment settings.

## 5 Conclusion

The Neoadjuvant Esophageal (NAE) score provides a robust and clinically meaningful tool for predicting survival and postoperative complications in patients with locally advanced ESCC undergoing neoadjuvant therapy and surgery. By outperforming conventional TNM staging and identifying high-risk patients who benefit from adjuvant therapy, the NAE score offers a practical approach to personalized treatment planning. Integration of this scoring system into clinical practice may optimize therapeutic strategies, improve risk stratification, and support decision-making in perioperative management. Further prospective multicenter studies are warranted to validate its utility across diverse settings and establish the NAE score as a standardized prognostic marker in ESCC. Given the study’s retrospective nature and lack of external validation, the NAE score should be considered a promising internally validated tool that requires external validation before widespread clinical implementation.

## Data Availability

The raw data supporting the conclusions of this article will be made available by the authors, without undue reservation.

## Supplementary material

Supplementary Figure 1 Kaplan–Meier graph for hospital length of stay in the matched patients by NAE score. Number at risk is shown below the plot.

Supplementary Figure 2 Kaplan–Meier graph comparing overall survival in low-NAE patients who received adjuvant systemic therapy versus no adjuvant systemic therapy. Number at risk is shown below the plot.

Supplementary Figure 3 Kaplan–Meier graph comparing overall survival in intermediate-NAE patients who received adjuvant systemic therapy versus no adjuvant systemic therapy. Number at risk is shown below the plot.

Supplementary Figure 4 Kaplan–Meier graph comparing overall survival in high-NAE patients who received adjuvant systemic therapy versus no adjuvant systemic therapy. Number at risk is shown below the plot.

Supplementary Figure 5 ROC curve of the nomogram for predicting postoperative Clavien–Dindo grade ≥ III complications. Area under the curve (AUC) = 0.789 (95% CI 0.738–0.840).

Supplementary Figure 6 DCA curve of nomogram for Clavien–Dindo grade ≥III complications.
