# Supplementary figures and images for: The neoadjuvant esophageal score: a prognostic tool for predicting survival and postoperative complications in esophageal squamous cell carcinoma
Source: Front Immunol. 2026 Jan 2;16:1706548. doi: 10.3389/fimmu.2025.1706548 (PMC12808431; doi:10.3389/fimmu.2025.1706548)

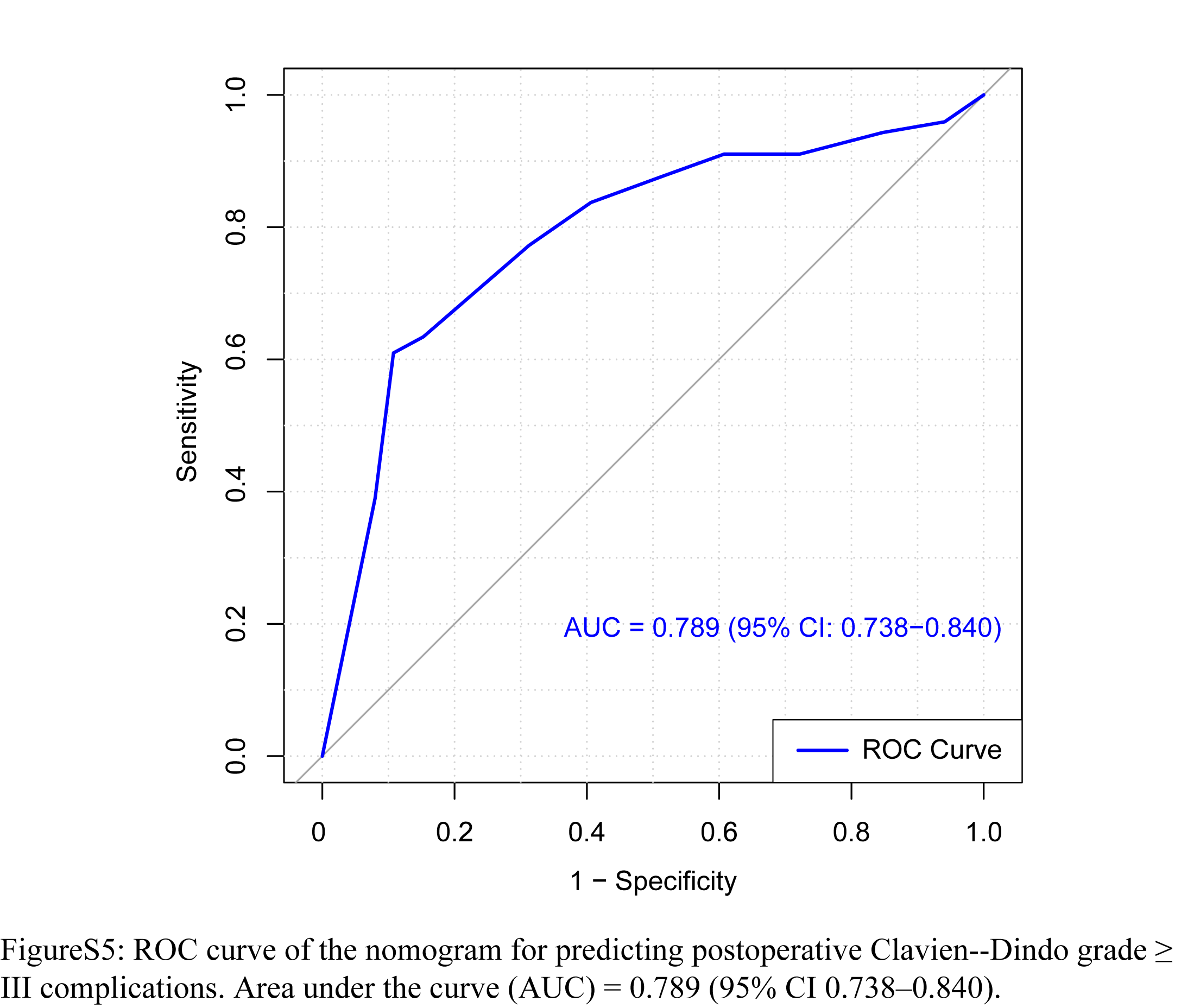

Supplement: Supplementary Figure 1 — Kaplan–Meier graph for hospital length of stay in the matched patients by NAE score. Number at risk is shown below the plot. [file Image1.tif]

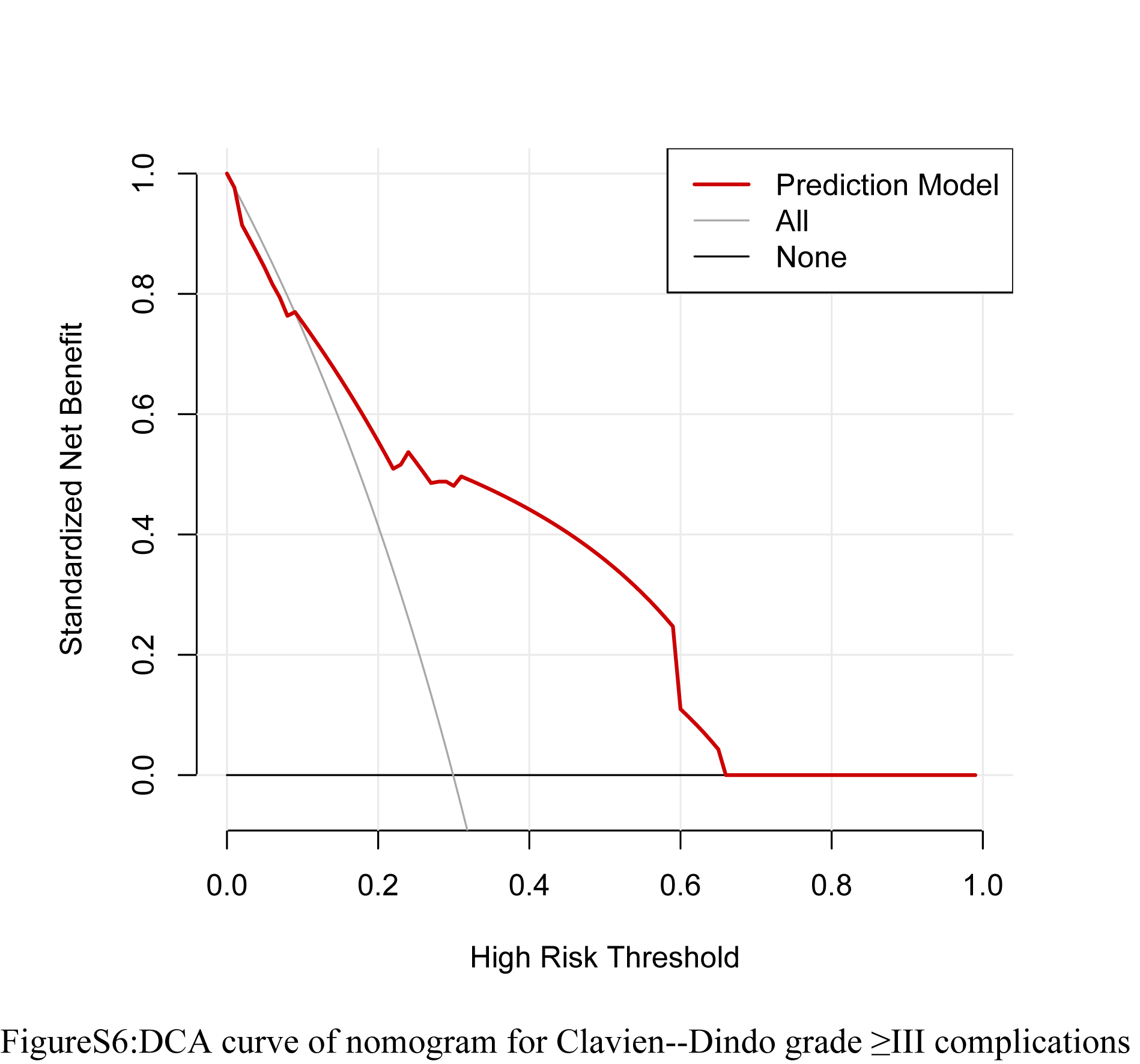

Supplement: Supplementary Figure 2 — Kaplan–Meier graph comparing overall survival in low-NAE patients who received adjuvant systemic therapy versus no adjuvant systemic therapy. Number at risk is shown below the plot. [file Image2.tif]

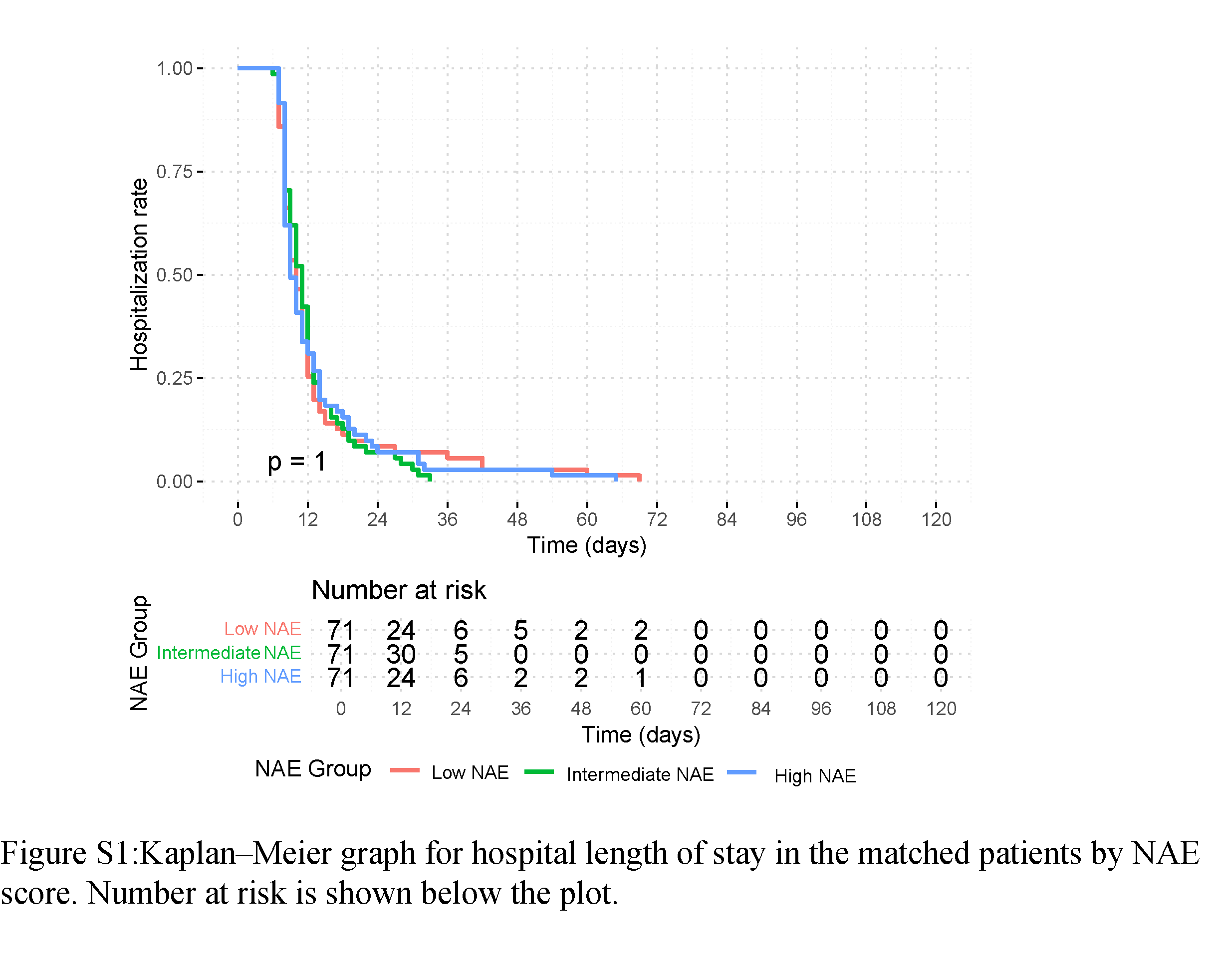

Supplement: Supplementary Figure 3 — Kaplan–Meier graph comparing overall survival in intermediate-NAE patients who received adjuvant systemic therapy versus no adjuvant systemic therapy. Number at risk is shown below the plot. [file Image3.tif]

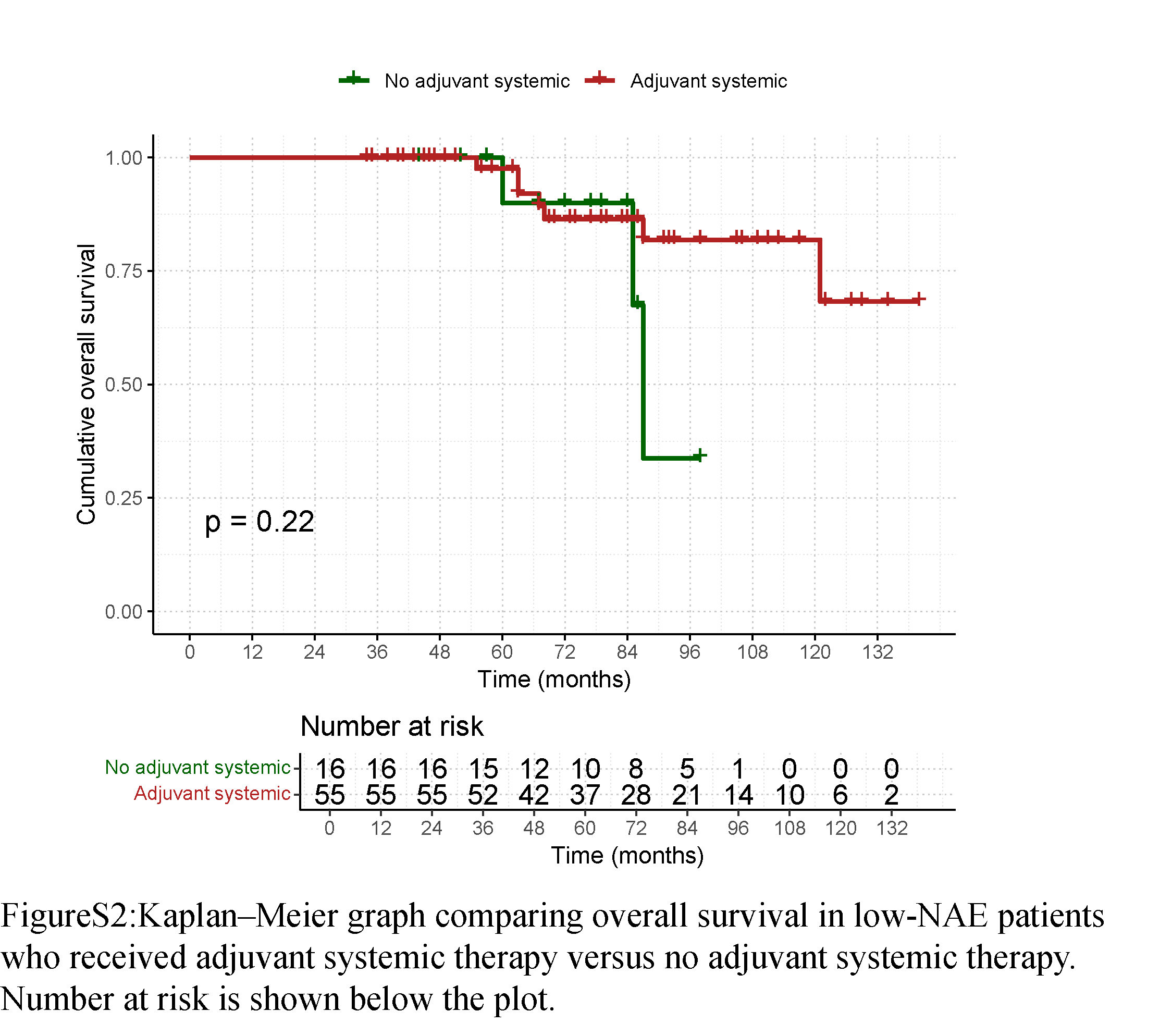

Supplement: Supplementary Figure 4 — Kaplan–Meier graph comparing overall survival in high-NAE patients who received adjuvant systemic therapy versus no adjuvant systemic therapy. Number at risk is shown below the plot. [file Image4.tif]

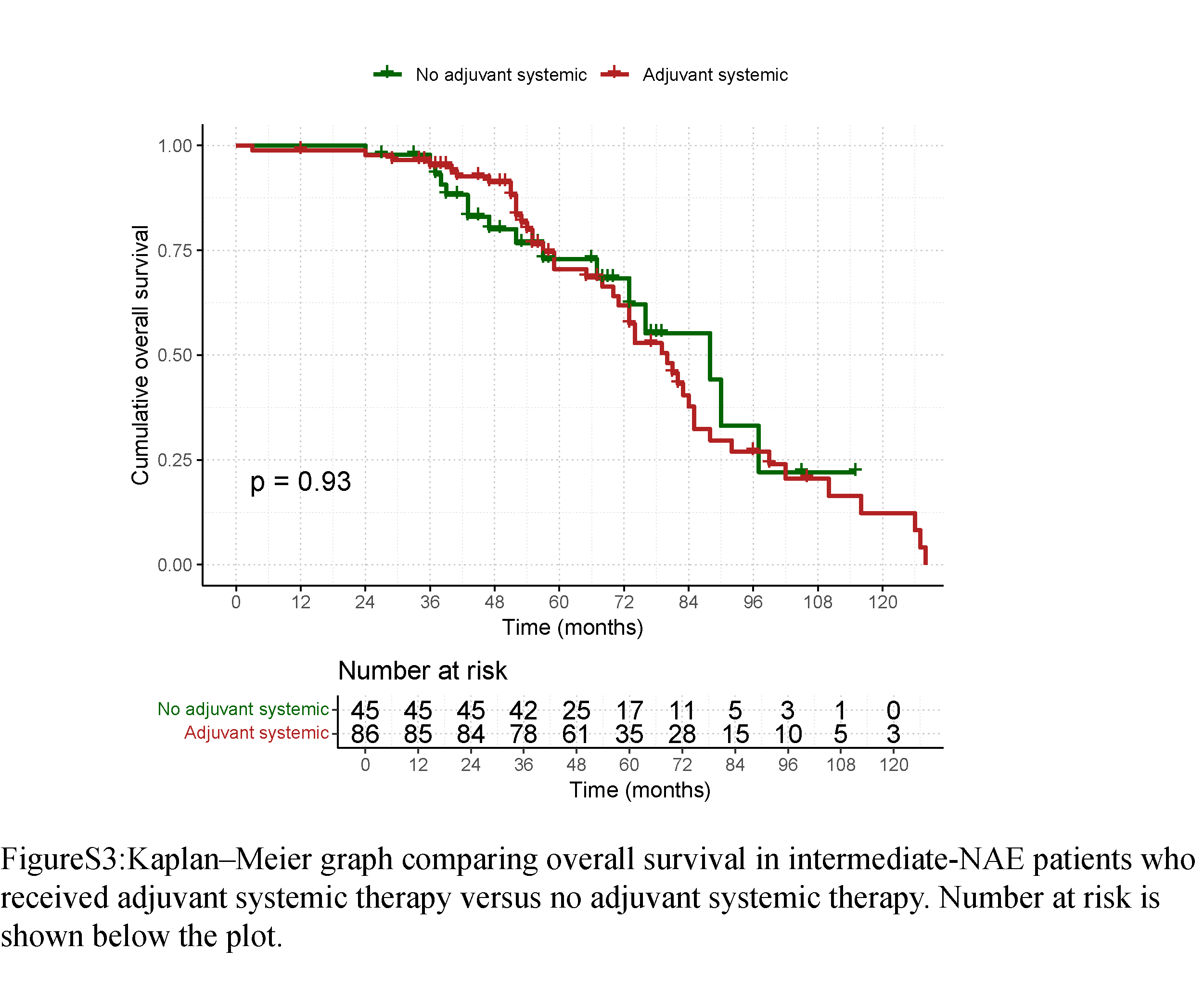

Supplement: Supplementary Figure 5 — ROC curve of the nomogram for predicting postoperative Clavien–Dindo grade ≥ III complications. Area under the curve (AUC) = 0.789 (95% CI 0.738–0.840). [file Image5.tif]

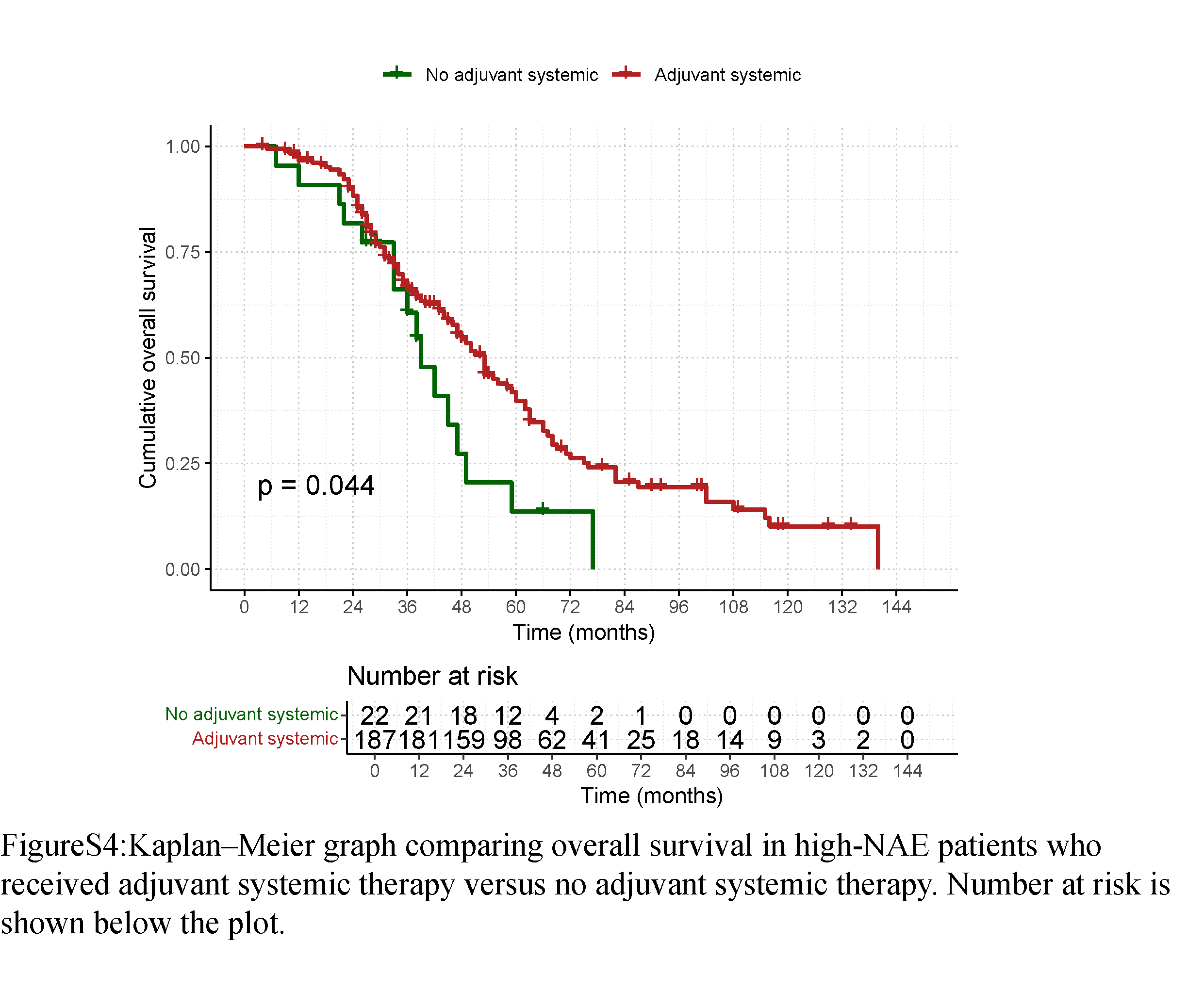

Supplement: Supplementary Figure 6 — DCA curve of nomogram for Clavien–Dindo grade ≥III complications. [file Image6.tif]
